# Molecular Mechanisms of Biofilm Infections and Combat Strategies

**DOI:** 10.3390/ijms25115823

**Published:** 2024-05-27

**Authors:** Honghua Hu

**Affiliations:** 1 Innovation Centre of Translational Pharmacy, Jinhua Institute, Zhejiang University, Jinhua 321016, China; honghuahu@zju.edu.cn; 2 College of Pharmaceutical Sciences, Zhejiang University, Hangzhou 310058, China

Microbial biofilms are the most important drivers of chronic and recurrent infections. Biofilms are associated with more than 80% of persistent infectious diseases, such as surgical implant infections, chronic wound infections, diabetic foot infections, periodontitis, chronic sinusitis, recurrent urinary tract infections, endocarditis, and chronic bronchitis pneumonia. The resistance of microbial cells in biofilms to the host immune system, antibiotics, and antimicrobial agents has increased hundreds of times compared to those in a planktonic state. Despite their clinical and scientific importance, the mechanisms of biofilm infections are still poorly understood, and the diagnosis and treatment of biofilm infections are challenging.

This Special Issue entitled “Molecular Mechanisms of Biofilm Infections and Combat Strategies” aims to understand the molecular basis, microbial community, pathogenicity, and host–pathogen interactions of biofilm infections to facilitate early intervention and the development of targeted therapeutic strategies. We received nine submissions for this Special Issue, six of which were published, including two review papers discussing the mechanisms of the Enterococcus genus and *Pseudomonas aeruginosa* biofilm formation; two original research papers evaluating the activities of two antibiofilm agents, namely ultrashort cyclic lipopeptides (USCLs) against *Candida albicans* biofilm and Anandamide against *Streptococcus mutans*; and two original research papers characterizing the properties of *Staphylococcus aureus* biofilms in different growth conditions.

Schiopu et al. presented a comprehensive review of the mechanisms in *E. faecalis* and *E. faecium* biofilm formation and explored potential eradication strategies. Biofilm formation in Enterococcus is a complex process that involves the interaction of various genes and virulence factors, such as gelatinase, cytolysin, Secreted antigen A, pili, microbial surface components, that recognize adhesive matrix molecules (MSCRAMMs), and DNA release. Quorum sensing is a critical mechanism mediating intercellular communication through peptide pheromones such as Cob, Ccf, and Cpd. It plays a vital role in coordinating biofilm development by regulating gene expression. In addition, extracellular DNA (eDNA) release is essential in biofilm formation. In *E. faecalis*, the autolysin N-acetylglucosaminidase and proteases such as gelatinase and serin protease play crucial roles in this process, affecting biofilm development and virulence. Targeting eDNA release may be a promising approach to combatting biofilm-producing E. faecalis infections.

Pulmonary infection with *P. aeruginosa* is a leading cause of morbidity and mortality in cystic fibrosis (CF) patients. The current antimicrobial treatments are inadequate due to the mode of growth of *P. aeruginosa* biofilm. A biofilm provides physical protection against antibiotics and creates niches, resulting in metabolic and phenotypic heterogeneity. Three biofilm-associated exopolysaccharides (EPSs) (alginate, Psl, and Pel) are being investigated as potential therapeutic targets for treating lung infection with *P. aeruginosa* in CF. Chung et al. reviewed the role of each EPS in the development and structure of *P. aeruginosa* biofilms and discussed emerging therapies and the barriers to their introduction to the clinic.

Czechowicz et al. aimed to determine the effectiveness of selected novel ultrashort cyclic lipopeptides (USCLs) and their combination with fluconazole against Vulvovaginal candidiasis (VVC). The researchers used an ex vivo model, which involved using tissue fragments of the mouse vagina, to study biofilm formation directly on the surface of the vaginal epithelium, and a BioFlux system, which assessed microorganisms (biofilms) in vitro under microfluidic to mimic in vivo conditions. The obtained results indicate the ineffectiveness of the tested substances ex vivo at concentrations that can eradicate biofilm in vitro, despite the combination of low concentrations of lipopeptides with mycostatic fluconazole showing promising results. The study revealed incompatibility between the classic in vitro methods, the BioFlux method, and the ex vivo method, highlighting the need for more research on the modification of standard methods for determining Candida susceptibility.

*S. mutans* is a bacterium that causes dental caries by forming plaque in the oral cavity. Wolfson et al. studied the effects of endocannabinoid anandamide (AEA), a naturally occurring bioactive lipid, on *S. mutans* viability, biofilm formation, and EPS production. AEA can eradicate *S. mutans* and prevent biofilm formation at 12.5 µg/mL. Moreover, AEA reduced the thickness and biomass of biofilms and lowered the total production of EPS. When tested on pre-existing biofilms, AEA’s impact on S. mutans biofilm at 50 µg/mL significantly increased the permeability of the membrane and induced hyper-polarization. The concentrations of AEA needed to induce anti-bacterial effects were below the cytotoxic concentration for normal Vero epithelial cells. The study suggests that AEA has anti-bacterial and anti-biofilm properties and potential as a therapeutic agent for preventing biofilms.

Recent findings regarding biofilms on dry hospital surfaces highlight failures in current cleaning practices [1] and the difficulty in removing these dry-surface biofilms (DSBs) [2]. Many aspects of complex DSB formation on environmental surfaces in healthcare settings are not fully understood. Rahman et al. used high-throughput tandem mass tag-based mass spectrometry to compare the proteins of *S. aureus* in 12-day dry-surface biofilms (DSB) and 12-day hydrated biofilms. The study revealed that significantly upregulated DSB proteins, compared to those of hydrated biofilms, are involved in peptidoglycan biosynthesis and are responsible for cell wall formation and thicker EPS matrix deposition, which may contribute to their persistence on dry surfaces. Parvin et al. further characterized the cell wall properties of *S. aureus* biofilms under different growth conditions. They discovered that the bacterial cell wall width and peptidoglycan production increased with the duration of biofilm culture and dehydration. The team also found that bacterial resistance to disinfectants was greatest in DSB, followed by 12-day hydrated biofilm and then 3-day biofilm, and lowest in planktonic bacteria. These findings suggest that the cell wall is an important factor in determining the biocide resistance of *S. aureus* biofilms, providing valuable insights that could help identify new targets to combat biofilm-related infections and hospital dry-surface biofilms.

We thank all the authors who contributed to this Special Issue and the reviewers for their valuable comments. We express our sincere gratitude to the editorial office of the International Journal of Molecular Sciences for their valuable support and for providing the opportunity to organize this Special Issue.
